# Effects of mis‐specified time‐correlated model error in the (ensemble) Kalman Smoother

**DOI:** 10.1002/qj.3934

**Published:** 2020-11-02

**Authors:** Haonan Ren, Javier Amezcua, Peter Jan van Leeuwen

**Affiliations:** ^1^ Department of Meteorology University of Reading Reading UK; ^2^ Department of Atmospheric Science Colorado State University Fort Collins Colorado USA

**Keywords:** data assimilation, linear model, model error, temporal auto‐correlation

## Abstract

Data assimilation is often performed under the perfect model assumption. Although there is an increasing amount of research accounting for model errors in data assimilation, the impact of an incorrect specification of the model errors on the data assimilation results has not been thoroughly assessed. We investigate the effect that an inaccurate time correlation in the model error description can have on data assimilation results, deriving analytical results using a Kalman Smoother for a one‐dimensional system. The analytical results are evaluated numerically to generate useful illustrations. For a higher‐dimensional system, we use an ensemble Kalman Smoother. Strong dependence on observation density is found. For a single observation at the end of the window, the posterior variance is a concave function of the guessed decorrelation time‐scale used in the data assimilation process. This is due to an increasing prior variance with that time‐scale, combined with a decreasing tendency from larger observation influence. With an increasing number of observations, the posterior variance decreases with increasing guessed decorrelation time‐scale because the prior variance effect becomes less important. On the other hand, the posterior mean‐square error has a convex shape as a function of the guessed time‐scale with a minimum where the guessed time‐scale is equal to the real decorrelation time‐scale. With more observations, the impact of the difference between two decorrelation time‐scales on the posterior mean‐square error reduces. Furthermore, we show that the correct model error decorrelation time‐scale can be estimated over several time windows using state augmentation in the ensemble Kalman Smoother. Since model errors are significant and significantly time correlated in real geophysical systems such as the atmosphere, this contribution opens up a next step in improving prediction of these systems.

## Graphical abstract

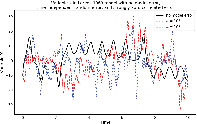

Variable *x* in the Lorenz 1963 model with no model error, time‐independent random error, and strongly auto‐correlated error.

## 1 INTRODUCTION

Data assimilation is a mathematical discipline to estimate the state of a system and its uncertainty by combining information from our prior knowledge of that system with observations of that system. From Bayes' theorem (Bayes, 1763), the general solution for the data assimilation problem is given by:

(1) $$p(x|y)=\frac{p(y|x)p(x)}{p(y)},$$

where **x** represents the state of the system, and **y** denotes the observations. The prior probability density function (pdf), *p*(**x**), contains the background information of the state variables, and the denominator *p*(**y**) is the marginal pdf of the observations and independent from state variables, and indeed plays no active role in state estimation. The conditional pdf *p*(**y**|**x**) contains the information from the observations, and is the probability density of the observations given the current state of the system. Lastly, the conditional pdf *p*(**x**|**y**) is the posterior which represents the probability of the state variable given the observations, and obtaining it is the ultimate goal of data assimilation. In the atmospheric and oceanic sciences, various approximate data assimilation methods have been developed in the past few decades, typically originating in either variational approaches (Courtier and Talagrand, 1987) or (ensemble) Kalman Filter‐based approaches (Evensen, 1994). Recently there has been a surge in hybrid methods trying to combine the advantages of the variational and KF‐based methods, for instance using variational methods to solve the ensemble problem (Zupanski, 2005).

In the past few decades, data assimilation methods like four‐dimensional variational method (4D‐Var) have often been performed under the assumption that the numerical models are perfect, known as the strong‐constraint setting (discussion in e.g., Amezcua and Van Leeuwen, 2018). Typically, it is assumed that the model errors can be neglected when compared with other error sources in the systems, such as the errors in the initial condition and observations (Trémolet, 2006). Since many dynamical systems of interest are chaotic, which means they are highly sensitive to the initial condition (Lorenz, 1963), a lot of research has focused on the errors in the initial condition in order to improve the accuracy of the weather forecast.

There are cases, however, when errors not coming from initial conditions become important in the accuracy of the forecasts and hence the data assimilation process. In fact, there is ample evidence that this is the case for most, if not all, geoscience disciplines (e.g., Bony *et al*., 2015; Kuma *et al*., 2018; Muelmenstadt and Feingold, 2018; Fox‐Kemper *et al*., 2019; Fennel *et al*., 2019; Fisher and Koven, 2020). These model errors are often hard to estimate, which has hampered their inclusion in the data assimilation process. However, there are many reasons why a proper estimate of model errors needs to be included, apart from the fact that they are there in our prediction models. Jazwinski (1970) points out that, in order to obtain an optimal estimate of the system, we need a better understanding of the error covariance matrices from all error sources. Furthermore, including random model errors in smoothers for chaotic systems such as the atmosphere and the ocean makes these system less dependent on initial conditions, allowing for more efficient optimisation and longer smoother windows. Indeed, with better understanding of initial and observational errors, and a strong reduction in the former, there has been an increasing number of works taking model errors into account in data assimilation process (e.g., Carrassi and Vannitsem, 2010; Howes *et al*., 2017), resulting in so‐called weak‐constraint data assimilation.

Model error is essentially the mismatch between the true evolution of the system and the forecast produced by the numerical model over one model time step. There are various sources for model errors in numerical models, such as numerical discretization of the underlying differential equations describing the system, incorrect parametrizations, missing physical processes, etc. Some works implement a random additive variable at any given time step as model error (e.g., Evensen and Van Leeuwen, 1996), or insert a random multiplicative factor in the tendencies of the model governing equations (Palmer *et al*., 2009). For simplicity, model errors are often considered Gaussian random variables with zero mean and no correlation over time. Alternatively, the model error can be considered to be fixed over the simulation period, resulting in a model bias. However, in operational systems, real model errors will be complex in both spatial and temporal behaviour, as can be inferred directly from the sources of these errors.

In this paper we study the case in which the spatial structure of the model error is known, but its temporal structure is uncertain. In reality, both space and time structure are unknown, but we focus on the latter. We consider that the nature run evolves with a true model error; that is, a random model forcing with a certain decorrelation time‐scale $\omega_{r}$. We label this time‐scale *memory*. The imperfect forecast model uses a guessed memory $\omega_{g}$, which is different from the real one.

**FIGURE 1:**
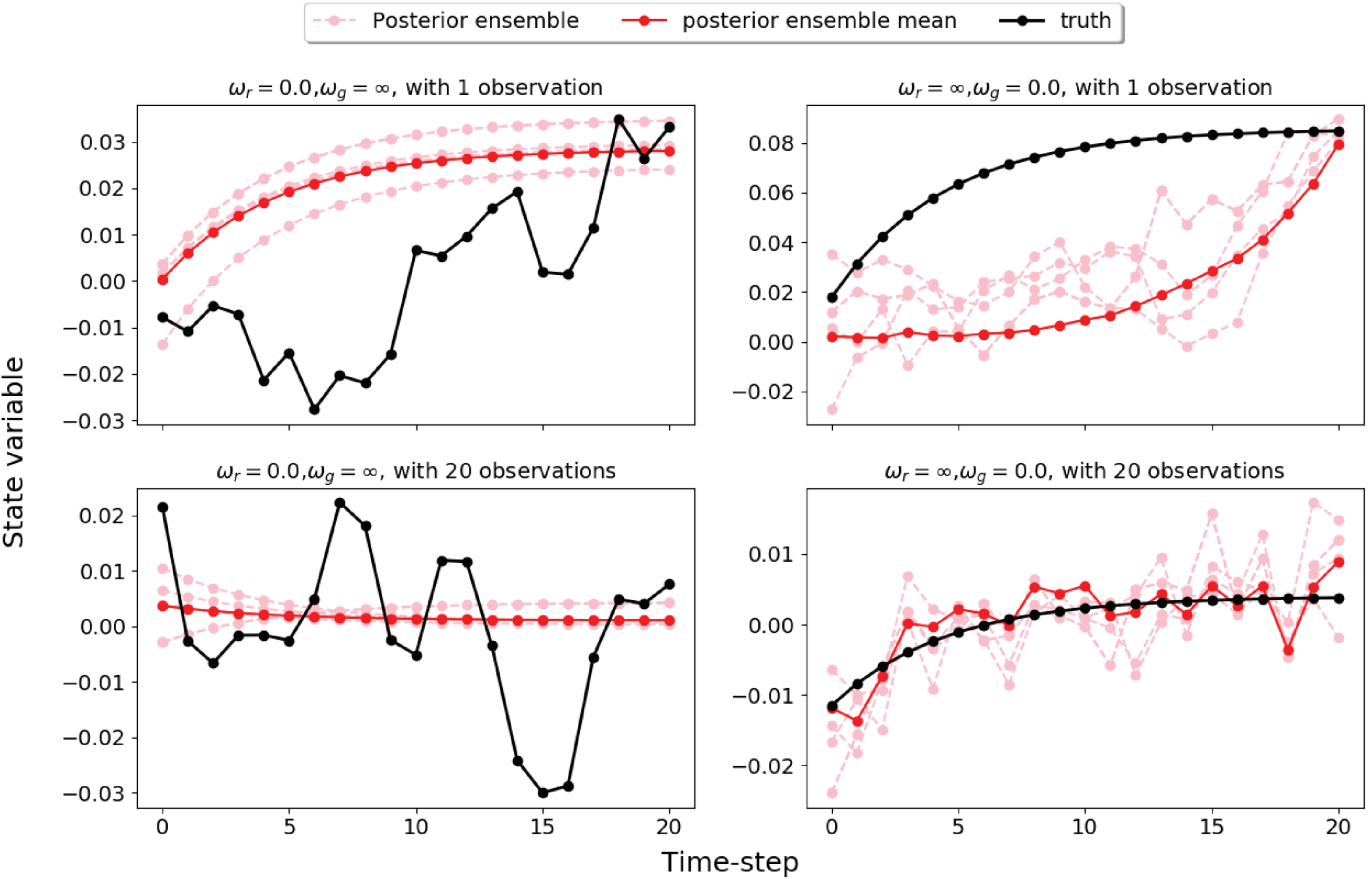
Plots of the trajectories of the true state of the system (black), three posterior ensemble members (pink, randomly chosen from 200 members), and the posterior ensemble mean (red). (a, c) show results for a true white‐noise model error and an assumed bias model error for two observation densities. Note that the posterior estimates are poor in both cases. (b, d) depict a bias true model error and an assumed white‐noise model error. The result with one observation is poor, while if many observations are present, the assimilation result is consistent within the ensemble spread [Colour figure can be viewed at wileyonlinelibrary.com]

This work has two main purposes. The first is to investigate the effect of the incorrect time‐correlated model error on data assimilation results under different observational frequencies, and different number of observations in an assimilation window. More specifically, we aim to quantify the change in performance of the Kalman Smoother when the time statistics of the model error are mis‐specified, and the sensitivity of this change to different assimilation parameters. These results are extended to the ensemble case. The second objective is to use the data assimilation process to diagnose the memory of the model error. This is of great importance since it allows us to discriminate between a bias and a completely time‐independent model error, and identify cases in between.

Before continuing, a simple illustration can illuminate the issue. In Figure 1 we show results of a smoothing process for a simple one‐dimensional system over a time window of 20 nature time steps. We use an ensemble Kalman Smoother with two different observation densities in time (the details are discussed in a later section). The memories in the nature model and the forecasts models do not coincide. We can see that with $\omega_{r}=0.0$, when the actual model error is a white‐in‐time random variable, the evolution of the true state of the system behaves rather randomly with the present model settings. If we do not know the memory and assume the model error is a bias in the data assimilation process ($\omega_{g}\to \infty$), the estimation made by the data assimilation method is not even close to the truth, even with very dense observations in the simulation period, as shown in Figures 1a, c. On the other hand, if the model error in the true model evolution behaves like a bias, and we assume that the model error is white in time in the data assimilation process, the results are quite different with different observation frequencies. As shown in Figures 1b, d, with very frequent observations, we can see a fairly good performance of the data assimilation process, but with a single observation the estimation is still not accurate.

The general structure of this paper is as follows. In section 2, we investigate the performance of the Kalman Smoother on a linear model with time‐correlated model error analytically. When we are unable to find closed expressions, we numerically evaluate the (open) analytical expressions when necessary. We determine the behaviour of the posterior variance and mean‐square error for different values of true and guessed memory. Next, a higher‐dimensional system is explored in Section 3 via numerical experiments using the Ensemble Kalman Smoother. In Section 4 we use state augmentation to try to infer the memory time‐scale from the assimilation process, with satisfactory results. Section 5 contains a summary and a discussion of the results.

In this paper we follow the notation introduced by Amezcua and Van Leeuwen (2018). Identifying different attributes in a variable can be difficult in some expressions. In general, superindices are used as time indices. If there is a comma in the superindex, it is because we have also added a label corresponding to the role in the data assimilation process. For instance, the variable $\mu_{x}^{t,b}$ would be the background mean of *x* at time *t*. There are some cases in which the function is clear, for instance a superscript applied to a vector cannot mean exponentiation. In the case where superscripts denote exponents, this is clearly specified in the text. An example of more complicated uses is: $K_{\omega_{g}}^{x,t}$ which refers to the Kalman gain evaluated in *x* space at time *t* computed with the covariance matrix which uses the guessed memory $\omega_{g}$. More complicated uses of the sub‐ and superscripts are clearly identified in the text, and we also recommend that the reader check Amezcua and Van Leeuwen (2018) for clarity.

## 2 TIME‐CORRELATED MODEL ERROR IN THE KALMAN SMOOTHER

Let us consider a simple linear model with the governing equation over one model time step:

(2) $$x^{t+1}=M^{t\to (t+1)}x^{t}+\nu^{t+1},$$

where $M^{t\to (t+1)}\in {ℛ}^{N_{x}\times N_{x}}$ represents the linear model operator and its size depends on the number of variables in the system *N*_*x*_, $x^{t}\in {ℛ}^{N_{x}}$ is the state variable at given time step *t*, and $\nu^{t+1}\in {ℛ}^{N_{x}}$ is an additive model error which contains correlation in time and space. The initial condition of the random variable, $x^{0}\in {ℛ}^{N_{x}}$, is drawn from a multivariate Gaussian distribution (MGD), $x^{0}∼𝒩(\mu_{x}^{\text{0,b}},B)$, where $\mu_{x}^{\text{0,b}}\in {ℛ}^{N_{x}}$ is the mean of the random variable and $B\in {ℛ}^{N_{x}\times N_{x}}$ is its covariance matrix.

The time‐correlated model error at time *t* also comes from a MGD, $\nu^{t}∼𝒩(0,Q)$, with zero mean and the covariance matrix $Q\in {ℛ}^{N_{x}\times N_{x}}$. We also consider spatial correlations for the model errors, hence **Q** is not diagonal. We follow Amezcua and Van Leeuwen (2018) and assume that the model errors are correlated in time as:

(3) $$\text{Cov}(\nu^{i},\nu^{j})=\phi (|i-j|,\omega )Q,$$

where ϕ(|i−j|,ω) represents the memory of the model errors, |*i* − *j*| is the absolute difference between time steps *i* and *j*, and ω represents the characteristic memory time‐scale of the model error. The function ϕ decreases monotonically to 0 as |*i* − *j*| increases, and the maximum value of the ϕ is 1.0 as the absolute difference between time steps *i* and *j* tends to 0. For simplicity, we choose an exponentially decaying memory for the model error:

(4) $$\phi (|i-j|,\omega )=\exp\left(-\frac{\left|i-j\right|}{\omega }\right).$$

When the correlation time‐scale ω tends to 0.0, which indicates no temporal correlation in model errors, the ϕ function becomes a Kronecker delta function and the linear model becomes a first‐order Markov model:

(5) $$\phi (|i-j|,\omega )=\left\{\begin{array}{cc} 1\; & \text{if}\;\;i=j,\\ 0\; & \text{otherwise}. \end{array}\right.$$

In the other limit when ω tends to infinity, the memory of the model errors becomes 1.0 and the model error is fixed in time.

### 2.1 Formulation of the Kalman Smoother

We start with the formulation of the Kalman Smoother as described in Amezcua and Van Leeuwen (2018). It uses an extended control variable, $z^{0:\tau }\in {ℛ}^{(\tau +1)\times N_{x}}$ over τ+1 model time steps. This construction simplifies the representation of the covariance matrix and the exposition of the method. This extended variable can be written as the initial state of the system $x^{0}\in {ℛ}^{N_{x}}$, plus a collection of the model errors over time, $\nu^{1:\tau }\in {ℛ}^{\tau \times N_{x}}$:

(6) $$z^{0:\tau }=\left[\begin{array}{c} x^{0}\\ \nu^{1:\tau } \end{array}\right].$$

The extended variables can be transformed back to state space via:

(7) $$x^{t}=M^{0:t}z^{0:t},$$

where $M^{0:t}\in {ℛ}^{(t+1)N_{x}\times N_{x}}$ is the extended model operator and can be formulated as a block‐matrix:

(8) $$M^{0:t}=\left[\begin{array}{c} M^{0\to t},M^{1\to t},M^{2\to t},M^{3\to t},\ldots \;,M^{(t-1)\to t},I \end{array}\right].$$

The extended form also follows a MGD $z^{0:\tau }∼𝒩(\mu_{z}^{0:\tau ,b},D^{0:\tau })$, with mean $\mu_{z}^{0:\tau ,b}\in {ℛ}^{(\tau +1)N_{x}}$:

(9) $$\mu_{z}^{0:\tau ,b}=\left[\begin{array}{c} \mu_{x}^{\text{0,b}}\\ \mu_{v}^{1:\tau ,b} \end{array}\right]=\left[\begin{array}{c} \mu_{x}^{\text{0,b}}\\ 0^{0:\tau } \end{array}\right].$$

In this case, the prior covariance matrix $D^{0:\tau }\in {ℛ}^{(\tau +1)N_{x}\times (\tau +1)N_{x}}$ has a simple form, which can be written as a block‐matrix:

(10) $$D^{0:\tau }=\left[\begin{array}{cc} B & 0\\ 0 & Q^{1:\tau } \end{array}\right].$$

The covariance matrix of the extended control variable has two separate and independent parts: the part that comes from the initial condition, $B\in {ℛ}^{N_{x}\times N_{x}}$, and the part that originates purely from the correlated model errors, $Q^{1:\tau }\in {ℛ}^{\tau N_{x}\times \tau N_{x}}$. The covariance matrix $Q^{1:\tau }$ is a block‐matrix and can be written as a Kronecker product of a *Toeplitz matrix* and the spatial covariance matrix of the model error, **Q**:

(11) $$Q^{1:\tau }=\Phi^{1:\tau }⊗Q,$$

where the Toeplitz matrix $\Phi^{1:\tau }\in {ℛ}^{\tau \times \tau }$ contains all the memory coefficients. This Toeplitz matrix, $\Phi^{1:\tau }$, has different forms in different scenarios:

- When ω→0, the Toeplitz matrix becomes an identity matrix, $I\in {ℛ}^{\tau \times \tau }$, and the Kronecker product $Q^{1:\tau }$ becomes a block‐diagonal matrix.
- When ω→∞, the Toeplitz matrix $\Phi^{1:\tau }$ is a matrix of ones and $Q^{1:\tau }$ becomes a block‐matrix, in which every block element is the spatial covariance matrix **Q**.

To demonstrate the structure of the Kalman Smoother solution, we consider only one single observation at time step τ. Details of the formulation with multiple observations can be found in Amezcua and Van Leeuwen (2018). Then, the Kalman gain acting upon the whole simulation period in extended‐variable space, $K_{z}^{0:\tau }$, can be computed as:

(12) $$K_{z}^{0:\tau }=D^{0:\tau }{\left(M^{0:\tau }\right)}^{T}H^{T}{\left\{HM^{0:\tau }D^{0:\tau }{\left(HM^{0:\tau }\right)}^{T}+R\right\}}^{-1}.$$

With the Kalman Gain, we can update the extended control variable using the Kalman equation, assuming that the state initial **x**^0^, the observation error η and the model error ν are statistically independent of each other. Hence, the analysis mean is:

(13) $$z^{0:\tau ,a}=z^{0:\tau ,a}+K_{z}^{0:\tau }d,$$

where **d** is the innovation between observations and the model output at observational time *t*, which can be calculated as:

(14) $$d=y-HM^{0:\tau }z^{0:\tau ,a}.$$

The vector **y** represents the observations obtained from the true evolution of the system by the observation operator, $H\in {ℛ}^{N_{y}\times N_{x}}$, including the observational error:

(15) $$y^{t}=Hx^{t,r}+\eta^{t},$$

where $\eta^{t}\in {ℛ}^{N_{y}}$ is the observational error which follows a zero‐mean MGD $\eta^{t}∼𝒩(0,R)$ and its size depends on the number of variables observed from the system *N*_*y*_, **x**^*t*, r^ represents the *real* state of the system at time step *t*, and $R\in {ℛ}^{N_{y}\times N_{y}}$ represents the covariance matrix of the observation errors. Note that the observational time can be anywhere inside the assimilation window 0≤t≤τ. Finally, the covariance matrix is updated via:

(16) $$A_{z}^{0:\tau }=(I-K_{z}^{0:\tau }HM^{0:\tau })D^{0:\tau }.$$

Considering more than one observation per assimilation window does not yield simple expressions. Instead, it can be done in two ways. First, we can consider modified expressions as in the appendix of Amezcua and Van Leeuwen (2018). Second, the observations can be assimilated serially one after the other. Since the observation error covariance matrix is assumed diagonal, this is equivalent to updating observations all‐at‐once.

### 2.2 Evaluating the performance of the Kalman Smoother with time‐correlated model error

Amezcua and Van Leeuwen (2018) established a framework to handle time‐correlated model errors in the Kalman Smoother and its ensemble implementation. Nonetheless, they did not evaluate the performance of the methods they discussed, and they did not study the consequences (in this performance) of using wrong memory of the model error in the forecast. This is one of the two new contributions of this work, and it is detailed in this section.

A data assimilation system should be able to produce accurate estimations of the posterior density function of the state variables. In practice, assuming a unimodal posterior, it should at least be able to produce a mean trajectory which remains “close" to the (unknown) truth, and provide an uncertainty measure corresponding to the true uncertainty of the mean with respect to the truth.

One common approach is to compare the root‐mean‐square error (RMSE) which is the true error of the posterior mean, with the posterior standard deviation, or spread, which is the error estimated by the data assimilation method (Fortin *et al*., 2014). When the data assimilation results give us the “best" estimation of the system, the ratio of the RMSE and the spread should approximately be equal to 1.0. To simplify the situation, instead of comparing the RMSE with the spread, we use the ratio of the mean‐square error (MSE) and the variance of the state variable.

Before proceeding to actual experiments, we find the analytical expressions for both the MSE of the background and analysis. We also analyse in detail the variance expressions shown in Amezcua and Van Leeuwen (2018). To simplify calculations, we assume that the state is one‐dimensional and the model operator is a damping coefficient, α. The model is pure noise if α tends to 0.0, and a random walk model when α=1.0. We choose a damping coefficient between 0.0 and 1.0 to ensure that the linear model is stationary. This leads to a model equation:

(17) $$x^{t+1}=\alpha x^{t}+\nu^{t+1}.$$

For the next subsections we work in the state variable space, that is, our control variable is $x^{0:\tau }$, for two reasons: the meaning of the expressions is more tractable, and the implementation in the ensemble case is more straightforward. The general expressions are obtained as double sums which are not easy to visualise. In some cases these double sums can be evaluated, leading to expressions provided in the Tables in the Appendix. In other cases we evaluate the expressions numerically and provide graphical illustrations.

#### 2.2.1 Posterior variance in the Kalman Smoother

The prior variance at any time and covariance between two different time steps in our scalar system have the following expressions:

(18) $$\begin{array}{ll} \mathrm{Var}(x^{t,b}) & =\alpha^{2t}b^{2}+q^{2}\overset{t}{\underset{i=1}{\sum }}\overset{t}{\underset{j=1}{\sum }}\alpha^{2t-i-j}\phi (|i-j|,\omega ),\;\\ \text{Cov}(x^{t_{1},b},x^{t_{2},b}) & =\alpha^{t_{1}+t_{2}}b^{2}+q^{2}\overset{t_{1}}{\underset{i=1}{\sum }}\overset{t_{2}}{\underset{j=1}{\sum }}\alpha^{t_{1}+t_{2}-i-j}\phi (|i-j|,\omega ), \end{array}$$

where *b*^2^ is the variance of the initial *x*^0^, the superscript b denotes the prior, and *q*^2^ is the variance of the model error. In (18), in the expressions involving the scalars α, *b* and *q*, the exponent actually means the constant raised to a power, as opposed to being a superscript.

According to (18), the prior covariance and variance have two sources: the initial condition which is the first term on the right‐hand side (RHS), and the auto‐correlated model errors as the double sum term on the RHS. Of course, (18) is only suitable for *t* > 0, *t*_1_ > 0, *t*_2_ > 0. As a special case, since the initial condition *x*^0,b^ is independent from the model errors at any given time, its variance and covariance are given by:

(19) $$\begin{array}{ll} \mathrm{Var}(x^{\text{0,b}}) & =b^{2},\;\\ \text{Cov}(x^{\text{0,b}},x^{t,b}) & =\alpha^{t}b^{2}. \end{array}$$

**FIGURE 2:**
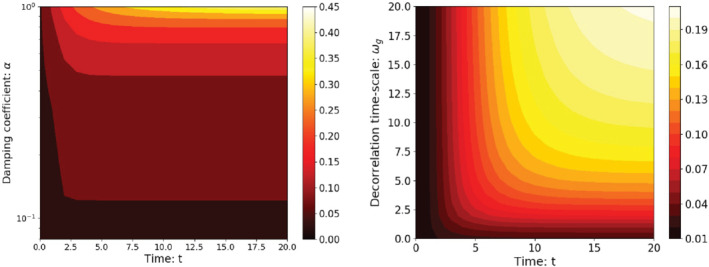
Prior variance as function of time in the window and (a) the damping coefficient α with $\omega_{g}=1.0$, and (b) time‐scale $\omega_{g}$ with α=0.8. Note that the *y*‐axis of (a) has a logarithmic scale [Colour figure can be viewed at wileyonlinelibrary.com]

Once more, the expression $\alpha^{t}$ means the constant α raised to the power *t*. To obtain a feeling for (18), Table .1 contains results on limiting cases for ω and α where the results of the sums can be evaluated analytically. Figure 2 shows that the prior variance is a monotonically increasing function of both α and $\omega_{g}$, and, not surprisingly, of time. The prior variance is almost constant when α<0.5. For larger values of α, the prior variance increases much faster with $\omega_{g}$. The prior variance as function of $\omega_{g}$ shows the opposite behaviour: when $\omega_{g}$ is between 0.0 and 10.0, the prior variance increases significantly with increasing $\omega_{g}$, but for larger $\omega_{g}$ values the increase of the variance slows down.

**TABLE 1:** Expressions for the prior variance, prior covariance and prior correlation in different scenarios

		α=0.0	0<α<1.0	α=1.0
ω=0.0	Var(*x*^*n*^)	*q*^2^	$\alpha^{2n}b^{2}+q^{2}\frac{\alpha^{2n}-1}{\alpha^{2}-1}$	*b*^2^ + *q*^2^*n*
	Cov(*x*^*n*^, *x*^*m*^)	$q^{2}\delta_{nm}$	$\alpha^{m+n}b^{2}+q^{2}\frac{\alpha^{m+n}-\alpha^{m-n}}{\alpha^{2}-1}$	*b*^2^ + *q*^2^*n*
	Corr(*x*^*n*^, *x*^*m*^)	$\delta_{nm}$	$\frac{\text{Cov}(x^{n},x^{m})}{\sqrt{\mathrm{Var}(x^{n})\mathrm{Var}(x^{m})}}$	$\sqrt{\frac{b^{2}+q^{2}n}{b^{2}+q^{2}m}}$
ω>0.0, but finite	Var(*x*^*n*^)	*q*^2^	$\alpha^{2n}+q^{2}\sum_{i=1}^{n}\sum_{j=1}^{n}\alpha^{2n-i-j}e^{\frac{-|i-j|}{\omega }}$	$b^{2}+q^{2}\sum_{i=1}^{n}\sum_{j=1}^{n}e^{\frac{-|i-j|}{\omega }}$
	Cov(*x*^*n*^, *x*^*m*^)	$q^{2}e^{\frac{-(m-n)}{\omega }}$	$\alpha^{m+n}+q^{2}\sum_{i=1}^{n}\sum_{j=1}^{m}\alpha^{m+n-i-j}e^{\frac{-|i-j|}{\omega }}$	$b^{2}+q^{2}\sum_{i=1}^{n}\sum_{j=1}^{m}e^{\frac{-|i-j|}{\omega }}$
	Corr(*x*^*n*^, *x*^*m*^)	$e^{\frac{-(m-n)}{\omega }}$	$\frac{\text{Cov}(x^{n},x^{m})}{\sqrt{\mathrm{Var}(x^{n})\mathrm{Var}(x^{m})}}$	$\frac{\text{Cov}(x^{n},x^{m})}{\sqrt{\mathrm{Var}(x^{n})\mathrm{Var}(x^{m})}}$
ω→∞	Var(*x*^*n*^)	*q*^2^	$\alpha^{2n}+q^{2}{\left(\frac{\alpha^{n}-1}{\alpha -1}\right)}^{2}$	*b*^2^ + *q*^2^*n*^2^
	Cov(*x*^*n*^, *x*^*m*^)	*q*^2^	$\alpha^{m+n}b^{2}+q^{2}\frac{\left(\alpha^{m}-1)(\alpha^{n}-1\right)}{{\left(\alpha -1\right)}^{2}}$	*b*^2^ + *q*^2^*nm*
	Corr(*x*^*n*^, *x*^*m*^)	1	$\frac{\text{Cov}(x^{n},x^{m})}{\sqrt{\mathrm{Var}(x^{n})\mathrm{Var}(x^{m})}}$	$\frac{b^{2}+q^{2}nm}{\sqrt{\left(b^{2}+q^{2}n^{2})(b^{2}+q^{2}m^{2}\right)}}$

Since the posterior variance is the estimated error resulting from the data assimilation scheme, and in linear data assimilation the posterior variance is independent of the actual value of the observations, the posterior variance has no knowledge of the real decorrelation time‐scale of the model errors, $\omega_{r}$. The posterior variance at a given time step, assuming that we have a single observation at time step τ, can be simplified as:

(20) $$\mathrm{Var}(x^{t,a})=\mathrm{Var}(x^{t,b})-K_{\omega_{g}}^{x,t}\text{Cov}(x^{\tau ,b},x^{t,b}),$$

where $K_{\omega_{g}}^{x,t}$ is the Kalman Gain formulated in the *x*‐space acting on the current time step *t* and in this scalar case can be computed as:

(21) $$K_{\omega_{g}}^{x,t}=\frac{\text{Cov}(x^{t,b},x^{\tau ,b})}{\mathrm{Var}(x^{\tau ,b})+r^{2}},$$

where *r*^2^ is the variance of the observation error, and clearly the exponent means the second power. We can see that the Kalman gain depends on the covariance between the state at the present time and at the observational time, the state variance at the observational time and the observation error. These expressions correspond to the state‐space formulation in Amezcua and Van Leeuwen (2018). We also compute some limiting cases on the posterior variance with a single observation for ω and α shown in Table .2.

**TABLE 2:** Expressions of the posterior variance in different scenarios

	α=0.0	0<α<1.0	α=1.0
ω=0.0	$q^{2}-\frac{q^{4}\delta_{t\tau }}{q^{2}+r^{2}}$	$\underset{\omega \to 0.0}{\lim}\mathrm{Var}(x^{t},x^{\tau })-\frac{\text{Cov}{\left(x^{t},x^{\tau }\right)}^{2}}{\mathrm{Var}(x^{\tau })+r^{2}}$	$(b^{2}+q^{2}t)-\frac{{\left(b^{2}+q^{2}t\right)}^{2}}{(b^{2}+q^{2}\tau )+r^{2}}$
0.0<ω≪∞	$q^{2}-\frac{q^{4}e^{\frac{-2(t-\tau )}{\omega }}}{q^{2}+r^{2}}$	$\mathrm{Var}(x^{t},x^{\tau })-\frac{\text{Cov}{\left(x^{t},x^{\tau }\right)}^{2}}{\mathrm{Var}(x^{\tau })+r^{2}}$	$\underset{\alpha \to 1.0}{\lim}\mathrm{Var}(x^{t},x^{\tau })-\frac{\text{Cov}{\left(x^{t},x^{\tau }\right)}^{2}}{\mathrm{Var}(x^{\tau })+r^{2}}$
ω→∞	$\frac{q^{2}r^{2}}{q^{2}+r^{2}}$	$\underset{\omega \to \infty }{\lim}\mathrm{Var}(x^{t},x^{\tau })-\frac{\text{Cov}{\left(x^{t},x^{\tau }\right)}^{2}}{\mathrm{Var}(x^{\tau })+r^{2}}$	$(b^{2}+q^{2}t^{2})-\frac{{\left(b^{2}+q^{2}t\tau \right)}^{2}}{b^{2}+q^{2}\tau^{2}+r^{2}}$

**FIGURE 3:**
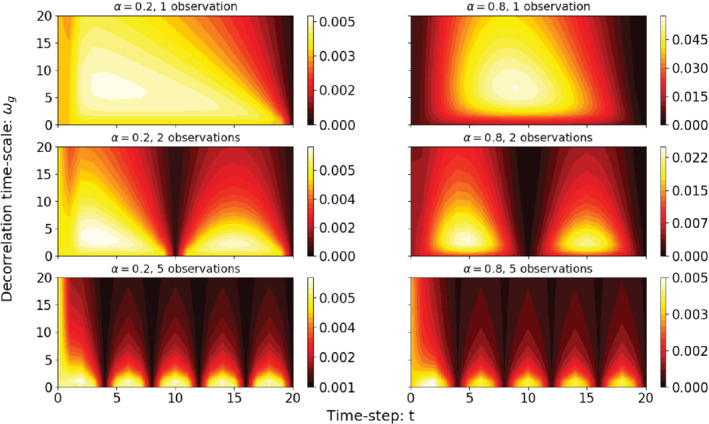
Posterior variance as function of time in the window and of $\omega_{g}$, for two fixed damping coefficients (a, c, e) α=0.2 and (b, d, f) α=0.8, using (a, b) one, (c, d) two, and (e, f) five observations in the simulation window. Note the different colour scales [Colour figure can be viewed at wileyonlinelibrary.com]

When more than one observation is present within an assimilation window, it is difficult to find simple analytical expressions and we refer to numerical evaluation. We start our numerical experiments with a fixed damping coefficient α=0.8, but with different memories ω. The results are shown in Figure 3.

The first thing that strikes the eye is the low posterior variance at observation times, which is as expected. Another clear trend is the decrease of posterior error with increasing $\omega_{g}$. This is directly related to the spread of observation information in the system: a larger $\omega_{g}$ gives more memory in the system, and hence observations have a larger influence over time. In some plots the posterior variance is decreasing towards the initial time, while in others it is increasing. However, this is mainly due to the different colour scales in the plots; the posterior variance at initial time is mainly set by the prior variance, although observations do have an influence for larger decorrelation time‐scales. Finally, one can notice a decrease of the posterior variance for $\omega_{g}$ close to zero. This behaviour has its roots in the behaviour of the prior, which has minimal variance for small $\omega_{g}$.

To see this latter point better, we resort back to the analytical treatment of the case of a single observation at the end of the simulation period, at t=τ. We focus on the posterior variance at the initial time and at the observational time as those times show most interesting behaviour. As we have seen above, the initial variance and the covariance between the initial state and the state at any time is independent of the decorrelation time‐scale, so:

(22) $$\frac{\partial \text{Cov}(x^{0,b},x^{t,b})}{\partial \omega_{g}}=\frac{\partial \mathrm{Var}(x^{\text{0,b}})}{\partial \omega_{g}}=0.$$

Using this, we find for the Kalman gain from (21) and Figure 2:

(23) $$\frac{\partial K_{\omega_{g}}^{x,0}}{\partial \omega_{g}}=-\frac{\partial \mathrm{Var}(x^{\tau ,b})}{\partial \omega_{g}}\frac{\text{Cov}(x^{\text{0,b}},x^{\tau ,b})}{{\left(\mathrm{Var}(x^{\tau ,b})+r^{2}\right)}^{2}}<0,$$

and so the Kalman gain for initial time is a decreasing function of the decorrelation time‐scale. Using this we find for the posterior variance at initial time:

(24) $$\frac{\partial \mathrm{Var}(x^{\text{0,a}})}{\partial \omega_{g}}=-\frac{\partial K_{\omega_{g}}^{x,0}}{\partial \omega_{g}}\text{Cov}(x^{\text{0,b}},x^{\tau ,b})>0,$$

which is an increasing function of the decorrelation time‐scale.

At the observational time we can do a similar derivation:

(25) $$\frac{\partial K_{\omega_{g}}^{x,\tau }}{\partial \omega_{g}}=\frac{\partial \mathrm{Var}(x^{\tau ,b})}{\partial \omega_{g}}\frac{r^{2}}{{\left\{\mathrm{Var}(x^{\tau ,b})+r^{2}\right\}}^{2}}>0,$$

leading to:

(26) $$\begin{array}{ll} \frac{\partial \mathrm{Var}(x^{\tau ,a})}{\partial \omega_{g}} & =(1-K_{\omega_{g}}^{x,\tau })\frac{\partial \mathrm{Var}(x^{\tau ,b})}{\partial \omega_{g}}-\frac{\partial K_{\omega_{g}}^{x,\tau }}{\partial \omega_{g}}\mathrm{Var}(x^{\tau ,b})\;\\ & =\frac{\partial \mathrm{Var}(x^{\tau ,b})}{\partial \omega_{g}}\frac{r^{4}}{{\left\{\mathrm{Var}(x^{\tau ,b})+r^{2}\right\}}^{2}}>0. \end{array}$$

We thus find that both at initial and at observation times, the posterior variance increases with $\omega_{g}$. In fact, this derivation shows that this is true for all values of $\omega_{g}$, at initial and final times, not only for small $\omega_{g}$ values as Figure 3 might suggest.

#### 2.2.2 Mean‐square error (MSE) of the posterior in the Kalman Smoother

Different from the posterior variance, for the MSE between the analysis mean and the true state of the system differences between the real decorrelation time‐scale and the one assumed in the data assimilation are important. We calculate the MSE of the prior as the difference between the prior mean $\mu^{t,b}$ and the truth. The truth is a realization of the true prior pdf at the initial time. The MSE at any time *t* is defined as:

(27) $${\text{MSE}}^{t,b}=\int {\left(\mu^{t,b}-x^{t,r}\right)}^{2}p(x^{t,r})\;dx^{t,r}.$$

When the statistics of the model error used in the data assimilation is different from that of the truth, the prior pdf used in the data assimilation will deviate from the pdf that the truth is drawn from. Writing the pdf from which the truth is drawn as $x^{t,r}\in 𝒩(\nu^{t},B^{t})$, where $\nu^{t}$ is its mean at time step *t* and *B*^*t*^ represents its variance, the MSE at time *t* becomes:

(28) $${\text{MSE}}^{t,b}=E_{x^{t,r}}\left[{\left(\mu^{t,b}-x^{t,r}\right)}^{2}\right]=B^{t}+{\left(\mu^{t,b}-\nu^{t}\right)}^{2},$$

in which the last term represents the bias in the prior. Using this in a Kalman Smoother, we can compute the posterior MSE as:

(29) $$\begin{array}{ll} {\text{MSE}}^{t,a} & =E_{x^{t,b}}\left[{\left(\mu^{t,a}-x^{t,b}\right)}^{2}\right]\;\\ & =E_{x^{t,b}}\left[(\mu^{t,b}-x^{t,r})+K_{\omega_{g}}^{x,t}(x^{\tau ,r}-\mu^{\tau ,b})+K_{\omega_{g}}^{x,t}{\left(y^{\tau }-x^{\tau ,r}\right)}^{2}\right]\;\\ & =B^{t}+{\left(\mu^{t,r}-\nu^{t}\right)}^{2}+{\left(K_{\omega_{g}}^{x,t}\right)}^{2}\left\{B^{\tau }+{\left(\mu^{\tau ,b}-\nu^{\tau }\right)}^{2}+r^{2}\right\}\;\\ & \;-2K_{\omega_{g}}^{x,t}\left\{\text{Cov}(x^{t,r},x^{\tau ,r})+(\mu^{t,r}-\nu^{t})(\mu^{\tau ,b}-\nu^{\tau })\right\}. \end{array}$$

In the ideal case when $\omega_{g}=\omega_{r}$, the MSE of the posterior can be simplified as:

(30) $$\begin{array}{ll} {\text{MSE}}^{t,a} & =B^{t}+{\left(K_{\omega_{g}}^{x,t}\right)}^{2}\left(B^{\tau }+r^{2}\right)-2K_{\omega_{g}}^{x,t}\text{Cov}(x^{t,r},x^{\tau ,r})\;\\ & =B^{t}-K_{\omega_{g}}^{x,t}\text{Cov}(x^{t,r},x^{\tau ,r}). \end{array}$$

As expected, the posterior MSE in the ideal case is the same as the posterior variance shown in (20) because the statistics of the prior and the truth are the same in this ideal case. When more than one observation is present in the time window, we can write the Kalman Smoother MSE as:

(31) $$\begin{array}{ll} {\text{MSE}}^{0:\tau ,a} & =E_{x^{0:\tau ,r}}[\mu^{0:\tau ,a}-x^{0:\tau ,r}]{\left[\mu^{0:\tau ,a}-x^{0:\tau ,r}\right]}^{T}\;\\ & =E_{x^{0:\tau ,r}}\left[\mu^{0:\tau ,b}+K_{\omega_{g}}^{x,0:\tau }\left(H^{1:L}x^{0:\tau ,r}\right.\right.\;\\ & \;\left.\left.-H^{1:L}\mu^{0:\tau ,b}+\eta^{0:\tau }\right)-x^{0:\tau ,r}\right]{\left[\ldots \right]}^{T}\;\\ & =E_{x^{0:\tau ,r}}\left[\left(I-K_{\omega_{g}}^{x,0:\tau }H^{1:L}\right)\left(\mu^{0:\tau ,b}\right.\right.\;\\ & \;\left.\left.-x^{0:\tau ,r}\right)+K_{\omega_{g}}^{x,0:\tau }\eta^{0:\tau }\right]{\left[\ldots \right]}^{T}\;\\ & =\left(I-K_{\omega_{g}}^{x,0:\tau }H^{1:L}\right)B_{\omega_{g}}^{0:\tau }\;\\ & \;+\left(I-K_{\omega_{g}}^{x,0:\tau }H^{1:L}\right)\left(\mu^{0:\tau ,b}-\nu^{0:\tau }\right)\;\\ & \;\times {\left(\mu^{0:\tau ,b}-\nu^{0:\tau }\right)}^{T}{\left(I-K_{\omega_{g}}^{x,0:\tau }H^{1:L}\right)}^{T}, \end{array}$$

where $\mu^{0:\tau ,a}$ is the time‐series of the posterior mean from the Kalman Smoother, $B^{0:\tau }$ represents the covariance matrix derived from the true pdf, $K_{\omega_{g}}^{x,0:\tau }$ is the Kalman Gain matrix calculated with $\omega_{g}$, and $\mu^{0:\tau ,b}$ is the prior mean time‐series. The observation operator **H**^1 : *L*^ maps *L* observations, from the state space into the observation space. Written in this form it is relatively easy to understand what the influence of a mis‐specified model error is. However, this is slightly deceiving in that the result is written in terms of the true covariance and mean, which are unknown in the real world, and the Kalman Gain using the incorrect model error description. In the ideal scenario the MSE can be simplified to:

(32) $${\text{MSE}}^{0:\tau ,a}=(I-K^{x,0:\tau }H^{1:L})\;B^{0:\tau },$$

where $K^{x,0:\tau }$ is the optimal gain for the Kalman Smoother. Equation (32) shows the exact solution for the posterior covariance matrix shown in (16) in the variable space.

**FIGURE 4:**
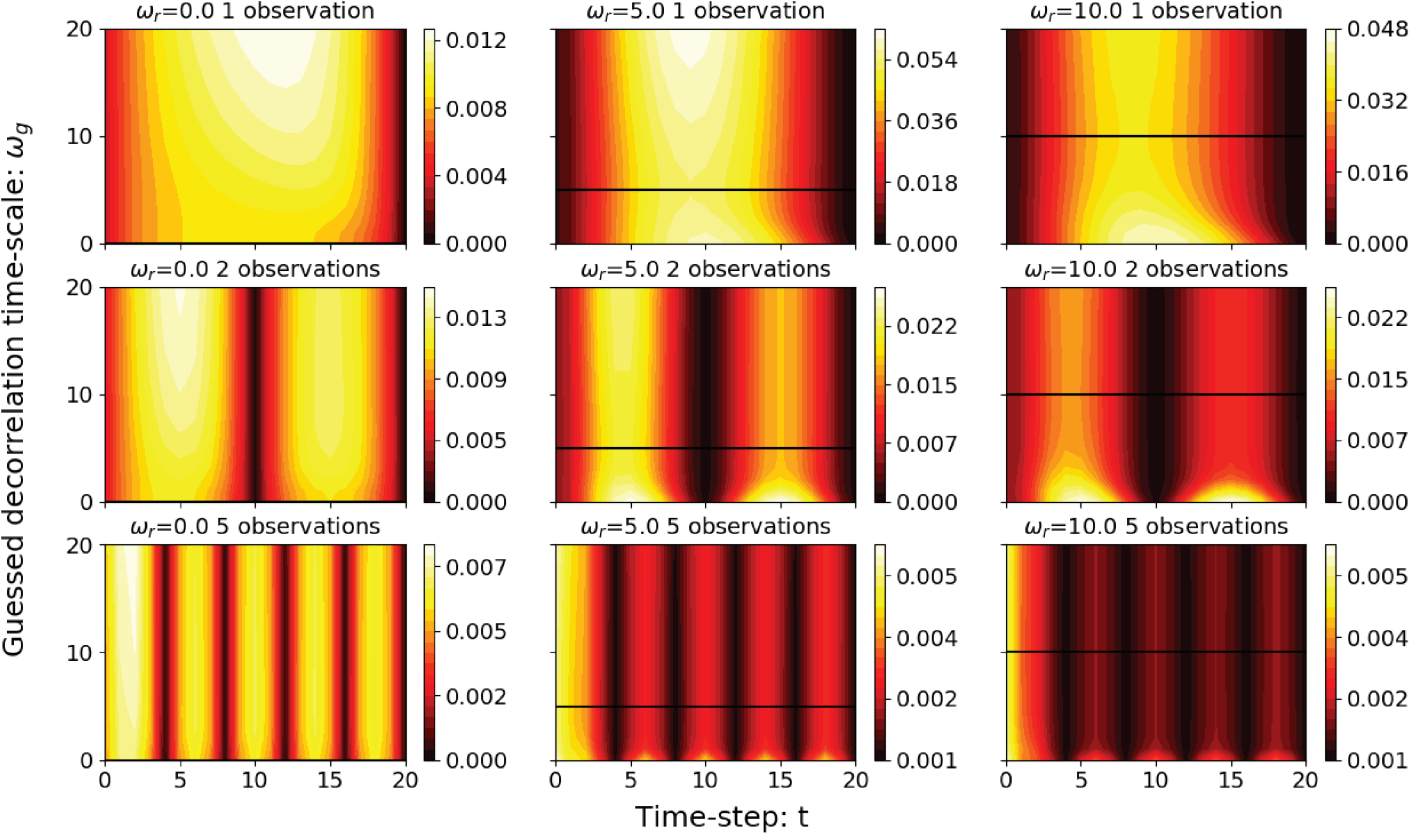
Posterior MSE as function of time in the window and of $\omega_{g}$, for different fixed $\omega_{r}=$ (a, d, g) 0, (b, e, h) 5, and (c, f, i) 10, and (a–c) one, (d–f) two and (g–i) five observations in the simulation window. The solid black line indicates where $\omega_{g}=\omega_{r}$ Note the different colour scales [Colour figure can be viewed at wileyonlinelibrary.com]

The behaviour of the posterior MSE when the memory in the prior differs with that of the true system, that is, $\omega_{g}\neq \omega_{r}$, is found from the numerical evaluation of the analytical expressions shown in (29), and the results are shown in Figure 4, which shows that, in general, the magnitude of the posterior MSE decreases as the observation frequency increases. This matches the results shown in Figure 3 for the posterior variance. As we expected, the posterior MSE reaches its minimum at the observational time. From Figure 4a,b,c, we can see that, with a single observation at the end of the simulation window, the MSE is minimized when $\omega_{g}=\omega_{r}$ for the time steps that are away from the observational time and initial state. However, when the number of observations in the window increases, the difference between $\omega_{r}$ and $\omega_{g}$ becomes less important: the solid lines do not dominate large changes in MSE.

The Appendix contains derivations and analytical results for the Ensemble Kalman Smoother, where we specifically study the influence of sampling errors.

### 2.3 Evaluation of the Kalman Smoother for a one‐dimensional system

**FIGURE 5:**
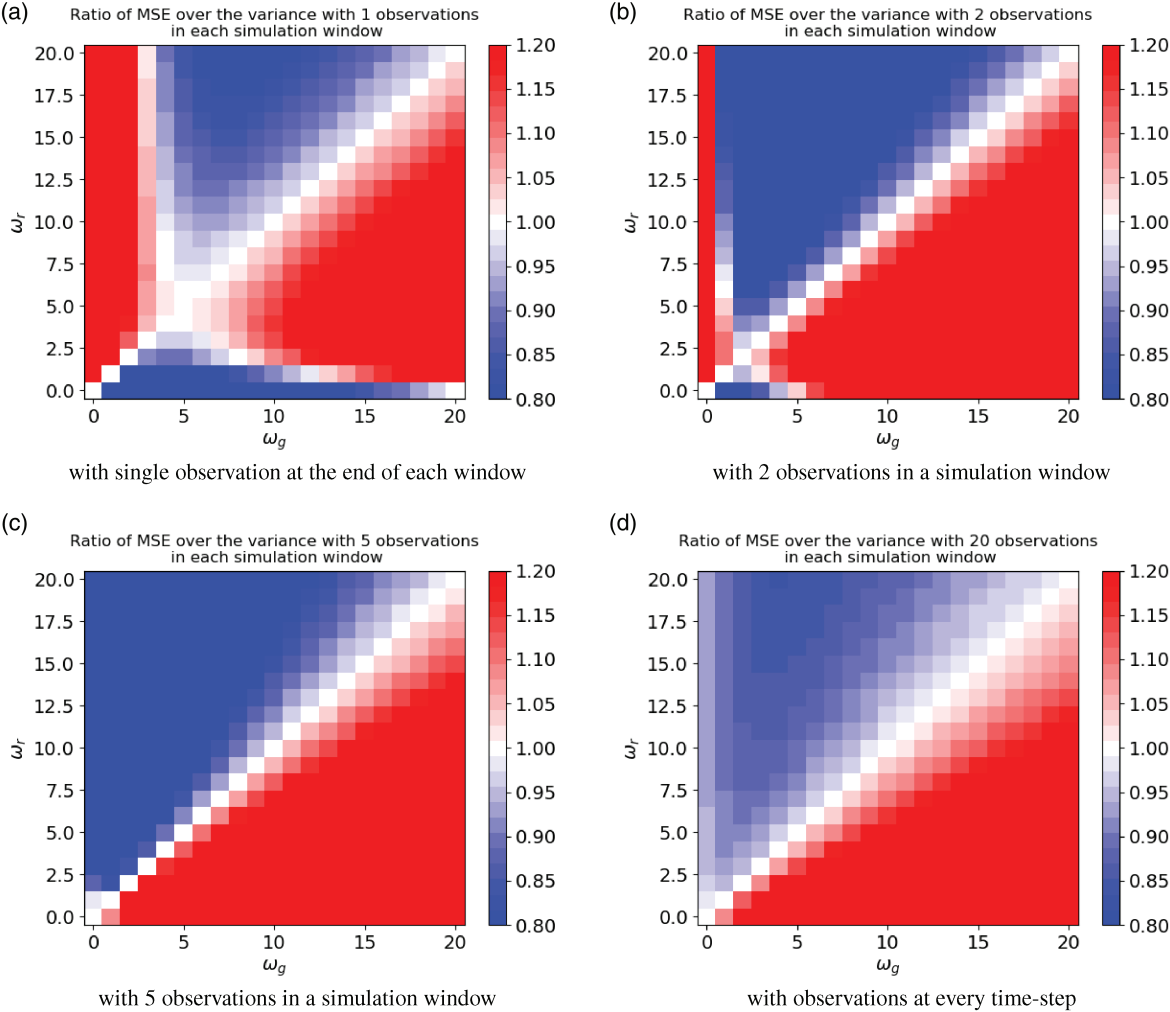
Ratio of MSE over the posterior variance for the one‐dimensional system, calculated using numerical evaluation of the exact analytical expressions, using (a) one, (b) two, (c) five, and (d) 20 observations [Colour figure can be viewed at wileyonlinelibrary.com]

To evaluate the performance of the Kalman Smoother we compute the ratio of the MSE over the variance of the posterior averaged over the simulation window, with different observational frequencies. Figure 5 shows the numerical evaluation of analytical expressions which contain ratios of double sums and are hence difficult to visualise without plotting them.

As we can see, the Kalman Smoother works well when $\omega_{g}=\omega_{r}$ for all the cases, with the ratio of MSE over the variance equal to 1.0. With relatively high observational frequency (five observations or more in the simulation window), the MSE is larger than the estimated posterior variance when $\omega_{g}>\omega_{r}$, and *vice versa*. From the numerical results shown Figure 4, the mismatch between the two time‐scales $\omega_{r}$ and $\omega_{g}$ barely has any impact on the MSE. The ratio is dominated by the posterior variance.

**FIGURE 6:**
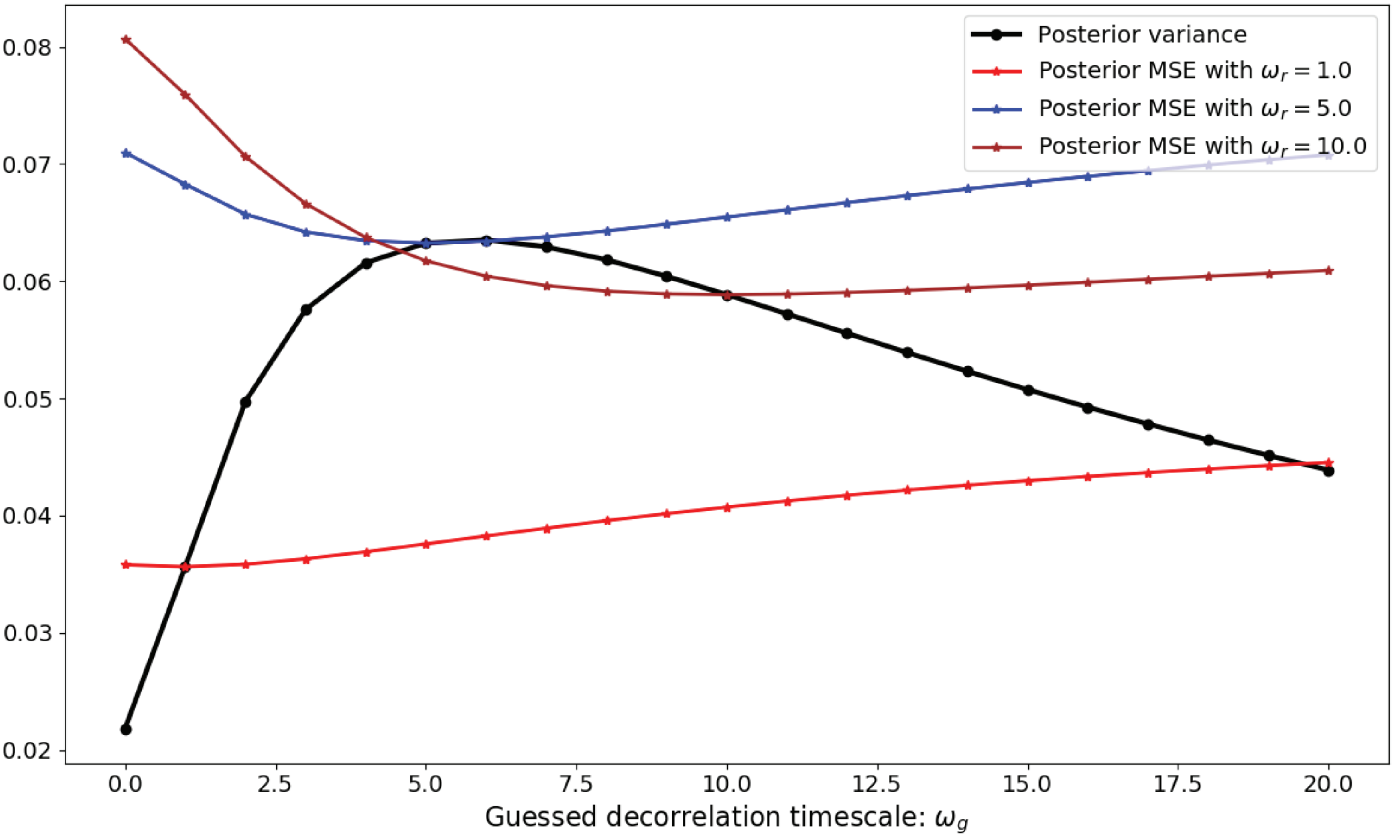
The time‐averaged posterior variance (black) and posterior mean‐square error with different $\omega_{r}$ (red,blue,brown), as a function of $\omega_{g}$, using a fixed damping coefficient α=0.8 [Colour figure can be viewed at wileyonlinelibrary.com]

To understand the behaviour of the ratio in Figure 5 for small observation numbers, we refer to Figure 6, which shows the time average posterior variance as function of $\omega_{g}$ for the case of one observation in the time window, as the black line. The concave shape is due to a combination of two effects. Firstly, the prior variance grows with $\omega_{g}$ as a larger $\omega_{g}$ gives rise to a larger decorrelation time‐scale, so errors persist in the time window. This effect leads to a growth in posterior variance with $\omega_{g}$. Secondly, a larger $\omega_{g}$ reduces the posterior variance because the larger decorrelation time‐scale allows the observation information to spread more over the time window. These two competing effects lead to a maximum in posterior variance.

Figure 6 also shows the MSE for three different values of $\omega_{r}$. The MSE curves are all convex, with a minimum when $\omega_{g}=\omega_{r}$, as expected since the minimum value of the MSE happens when the guess decorrelation time‐scale is equal to the real time‐scale. The ratio the MSE to the posterior variance is equal to one when their curves cross, and we see immediately that the curves cross twice when the position of the minimum of the MSE is different from that of the maximum in the posterior variance. This is exactly what Figure 5 shows for one observation, and the structure of that solution is fully determined by the position of the peak in the posterior variance. For two observations, we see qualitatively similar behaviour, with the peak in the posterior variance shifting closer to zero. For 5 and 20 observations, the peak in the posterior variance shifts all the way to zero because the influence of the observations becomes more important than the prior, so the posterior variance becomes a decreasing function of $\omega_{g}$, as can also be observed in Figure 3. This means that the MSE and posterior variance curves only cross once, where $\omega_{g}=\omega_{r}$, as Figure 5 indeed shows.

Ideally we would be able to show this behaviour exploring the analytical expressions of (18), but the expressions become rather complicated as we would have to analytically evaluate ratios of integrals over double sums, which we were unable to perform. Finally, it should be noted that Figure 5 first calculates the MSE over posterior variance ratio and then averages over time, while Figure 6 and the argument above first average over time and then calculate the ratio. The results are qualitatively the same because Figures 3 and 4 show a similar behaviour over time.

## 3 TIME‐CORRELATED MODEL ERROR IN A HIGHER‐DIMENSIONAL SYSTEM

In this section we explore how the analytical results from the one‐dimensional system carry over to systems with relatively higher dimensions. To this end we implement an Ensemble Kalman Smoother (EnKS; spiciteEvensen2000ensemble) using perturbed model forecasts (Van Leeuwen, 2020) with 200 ensemble members on a ten‐dimensional system in which the deterministic model consists of a diagonal matrix with the damping coefficient on the diagonal, and spatially and temporally correlated model errors. This means that, although the elements of the state are evolving independently over time, they become more and more correlated due to the correlated model error. The large ensemble size with respect to the size of the state variable ensures that sample effects are small. Four cases with four different observation frequencies are discussed, similar to the experiments we do for the one‐dimensional system.

We generate the true trajectory of the system, $x^{0:\tau ,r}$ with $\omega_{r}$, and all the prior ensemble members are generated using $\omega_{g}$. The assimilation is run over 50 time windows, in which the results from one window provide the prior for the initial conditions for the next window (i.e., cycling). There are 20 time steps (τ=20) in each time window.

We experiment with different combinations of $\omega_{r}$ and $\omega_{g}$ with the same range from 0.0 to 20.0, and the four observation settings explored above. To evaluate the performance on the EnKS, we calculate the ratio of the MSE and the ensemble variance. The MSE at a given time step *t* is computed as:

(33) $${\text{MSE}}^{t}=\frac{{\left(x^{t,r}-{\bar{x}}^{t,a}\right)}^{T}(x^{t,r}-{\bar{x}}^{t,a})}{N_{x}},$$

where ${\bar{x}}^{t,a}$ is the mean of the posterior ensemble and **x**^*t*, r^ is the true state of the system.

**FIGURE 7:**
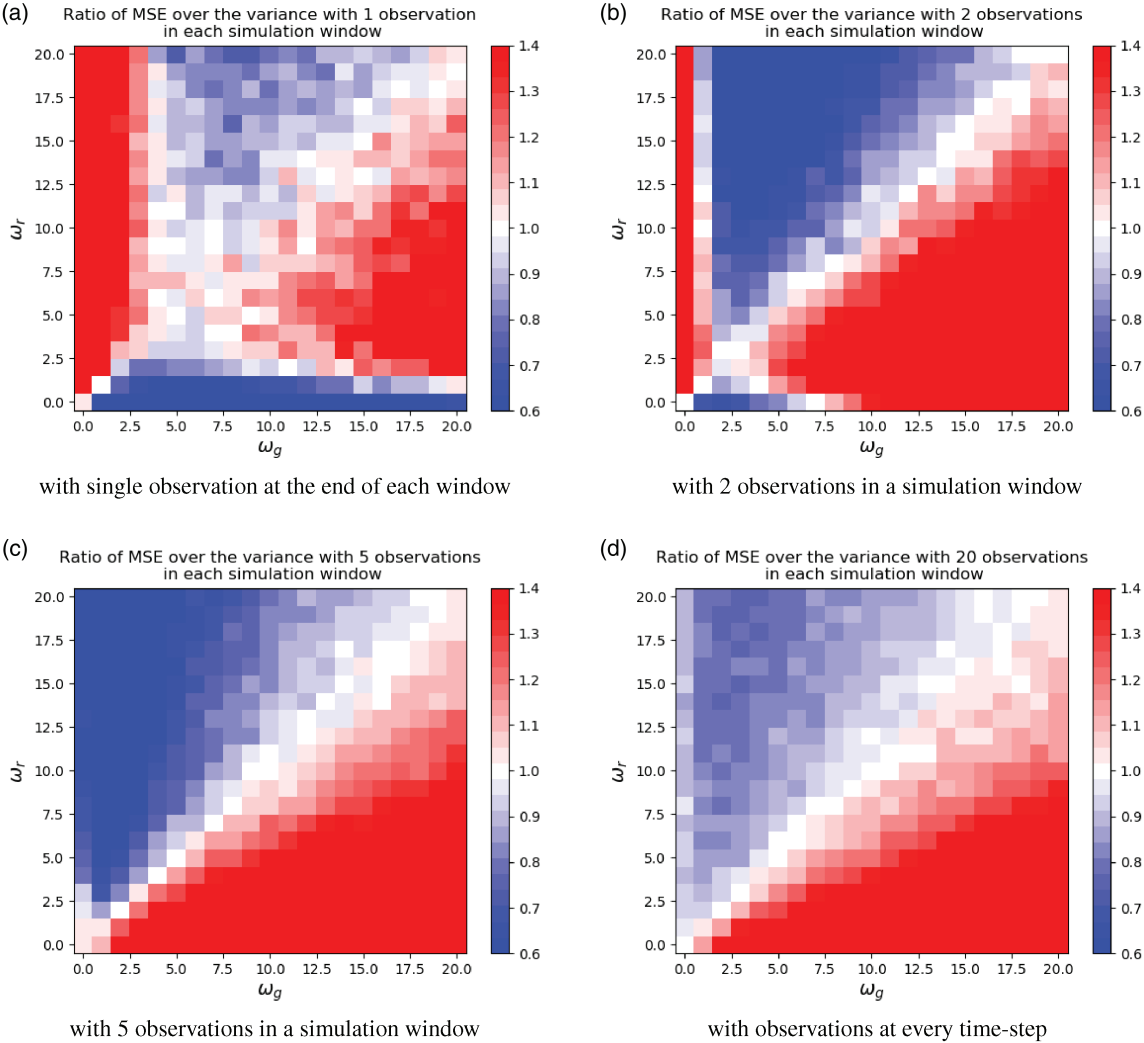
Ratio of MSE over the variance of the posterior for a ten‐dimensional system with (a) one, (b) two, (c) five, and (d) 20 observation frequencies in each simulation window. These plots come from numerical experiments with a 200‐member EnKS. Note that the results are qualitatively and also quantitatively very similar to those in Figure 5 [Colour figure can be viewed at wileyonlinelibrary.com]

After obtaining the MSE and the variance for each time step, we calculate their ratio and the average of this ratio over the whole simulation period, and the results are shown in Figure 7. We find that the ratio of MSE over the posterior variance matches with the results for one‐dimensional system shown in Figure 5. The EnKS performs well with a correctly guessed decorrelation time‐scale ($\omega_{g}=\omega_{r}$). With different observation frequencies, the ratio shows a similar behaviour as the ratio of MSE over the variance of the posterior in the one‐dimensional system, but the differences are about 10% larger in the higher‐dimensional case. For the higher‐dimensional system, it seems that the posterior ensemble spread is still the main factor to the ratio, which has a non‐monotonic behaviour with $\omega_{g}$ and becomes monotonically decreasing as $\omega_{g}$ increases.

## 4 ESTIMATION OF THE MEMORY IN THE MODEL ERROR

In the previous sections we showed that using an incorrect memory time‐scale in the model error can have a significant impact on the data assimilation results. Unfortunately, in many practical situations we do not know this memory time‐scale. However, it is possible to treat that correlation time‐scale as an unknown quantity and perform parameter estimation along the state estimation.

Parameter estimation via state augmentation has been used before, for example, with an extended Kalman Filter (Carrassi and Vannitsem, 2011), and even to determine parameters in Lagrangian Data Assimilation (Kuznetsov *et al*., 2003). Evensen (2009) used the EnKF to update the state while performing the parameter estimation.

Even for the simple linear regressive model that we used in the previous section, since the correlation time‐scale is deeply encoded inside the governing equation of the system, parameter estimation becomes a nonlinear problem. As an example of such a correlation time‐scale estimation problem, we will use state augmentation in an EnKS, in which the time‐scale is simply added to the state vector.

**FIGURE 8:**
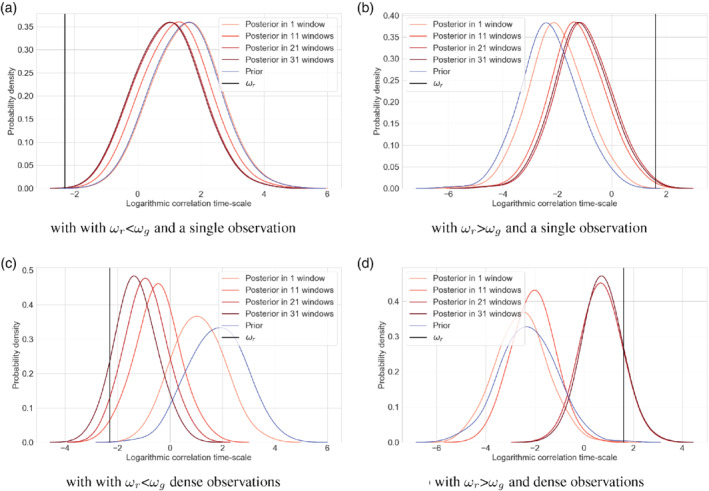
PDFs of the prior (blue) and posterior (reddish colours) estimated $\omega_{g}$, using an increasing number of assimilation windows. The different panels show results for different observation densities and prior mean (a, c) larger or (b, d) smaller than $\omega_{r}$. The vertical black line denotes the true value $\omega_{r}$ [Colour figure can be viewed at wileyonlinelibrary.com]

Instead of the memory time‐scale, $\omega_{g}$, we use the log scale of the memory time‐scale to avoid negative memory estimates. The initial log‐timescale values are drawn from a normal distribution: $\ln{\omega_{g}}_{i}\in 𝒩(\ln\omega_{g},1.0)$. Hence we assume that the prior distribution of the memory time‐scale is log‐normal distributed. The results are shown in Figure 8. Figure 8a, b show experiments with only one observation at the end of the window, in which either our first estimate of the time‐scale is larger or smaller than the real time‐scale. With an increasing number of windows, we obtain better estimates, but the variance of the estimate does not change. Also, the convergence is slow. We experimented with different values for first guess and true time‐scales, and in some cases the solution did not converge to the correct value. This is not surprising given the highly nonlinear character of the parameter estimation problem, especially with only one observation per window.

When we observe every time step, the convergence is much faster, and the variance in the estimate decreases, as shown in Figure 8c, d. In this case we always found fast convergence with different first guess and true time‐scale combinations, demonstrating that more observations bring us closer to the truth, and hence make the parameter estimation problem more linear.

## 5 CONCLUSION

In this paper we have investigated the influence of a mis‐specified model error decorrelation time‐scale in linear models, using an (Ensemble) Kalman Smoother, and investigated estimation of that time‐scale in an EnKS.

Using a Kalman Smoother, analytical results were derived for the posterior variance and Mean‐Squared Error (MSE) for a zero‐dimensional model. We find that the posterior variance, which only depends on the guessed correlation time‐scale $\omega_{g}$, has different behaviour with different observation frequencies. With a single observation, the posterior variance has a maximum at a certain $\omega_{g}$ value, and that maximum and the $\omega_{g}$ value decrease over time. When we increase the number of observations, the posterior variance becomes a monotonic decreasing function of $\omega_{g}$. Since the posterior variance represents the error of the posterior estimated by the data assimilation process, with more information from the observations the estimated error becomes significantly smaller. The MSE, which is the actual error of the posterior mean, decreases as well when more observations are included. But, unlike the posterior variance, the MSE of the posterior mean does not only depend on $\omega_{g}$, but also on the real memory time‐scale $\omega_{r}$. The results for the posterior MSE with a single observation show that it increases with both $\omega_{r}$ and the mismatch between $\omega_{g}$ and $\omega_{r}$. It means that, if we do not have a fair estimate of the correlation time‐scale, the actual posterior error will be larger.

For a higher‐dimensional model we used an EnKS. The results agree with the results from the analytical and numerical evaluations of the Kalman Smoother. For many observations we found that the MSE is larger than the estimated error for $\omega_{g}>\omega_{r}$, and *vice versa*. For a low number of observations, a new regime appears where for very small $\omega_{r}$ the MSE is smaller than the estimated error, and vice versa for very small $\omega_{g}$. This behaviour is mainly dictated by the behaviour of the estimated error.

Since the influence of an incorrect decorrelation time‐scale in the model error can be significant, we investigated the estimation of this time‐scale within an EnKF. We found that, when the observation density is high, state augmentation is sufficient to obtain converging results. However, with only one observation in a time window, the problem becomes too nonlinear and the estimation process is slow, or does not even converge. These results are consistent with parameter estimation via state augmentation in the literature. The new element is that online estimation is possible beyond a relatively simple bias estimate of the model error.

As a next step we will explore the influence of incorrectly specified model errors in nonlinear systems, with the goal to come up with a robust estimation method for time‐correlated model errors.

## APPENDICES

The prior variance, prior covariance and prior correlation

The posterior variance with a single observation at the end of the window

The Mean‐Square Error in the finite ensemble and scalar case

Let us start with the simplest case for the finite ensemble member size with only one observation at t=τ, with the ensemble members having the same distribution as the truth and hence the same model‐error memory. The ensemble size is *N*_e_, and the ensemble members $\left\{x_{1}^{t,b},x_{2}^{t,b},...,x_{N_{e}}^{t,b}\right\}$ and the truth are drawn from $𝒩(\mu^{t,b},{B^{t}}^{2})$. The sample mean, ${\bar{x}}_{N_{e}}^{t,b}$, has the distribution $∼𝒩(\mu^{t,b},{B^{t}}^{2}\;/N_{e})$, and the MSE of the prior sample mean is given by:

(34) $$\begin{array}{ll} E_{x^{t,r}}\left[{\left({\bar{x}}_{N_{e}}^{t,b}-x^{t,r}\right)}^{2}\right] & =E_{x^{t,r}}\left[{\left\{({\bar{x}}_{N_{e}}^{t,b}-\nu^{t,b})-(x^{t,r}-\nu^{t,b})\right\}}^{2}\right]\;\\ & =E_{x^{t,r}}\left[{\left({\bar{x}}_{N_{e}}^{t,b}-\nu^{t,b}\right)}^{2}\right]+E_{x^{t,r}}\left[{\left(x^{t,r}-\nu^{t,b}\right)}^{2}\right]\;\\ & =\frac{{B^{t}}^{2}}{N_{e}}+{B^{t}}^{2}. \end{array}$$

As we can see, the prior MSE under the perfect assumption is just the variance of the truth if *N*_e_ → *∞*. In this case, the posterior MSE of the ensemble mean can be computed as:

(35) $$\begin{array}{ll} E_{x^{t,r}}\left[{\left({\bar{x}}_{N_{e}}^{t,a}-x^{t,r}\right)}^{2}\right] & =E_{x^{t,r}}\left[{\left\{{\bar{x}}_{N_{e}}^{t,b}+K_{\omega_{r}}^{x,t}(\bar{y}-H{\bar{x}}_{N_{e}}^{\tau ,t})-x^{t,r}\right\}}^{2}\right]\;\\ & ={B^{t}}^{2}-K_{\omega_{r}}^{x,t}\text{Cov}(x^{t,r},x^{\tau ,r})+\frac{{B^{t}}^{2}}{N_{e}}\;\\ & \;-K_{\omega_{r}}^{x,t}\text{Cov}({\bar{x}}_{N_{e}}^{t,b},{\bar{x}}_{N_{e}}^{\tau ,b}). \end{array}$$

Even with the ideal assumptions, the posterior MSE for the finite ensemble case is not as simple as the prior MSE. The first two terms on the RHS represents the real MSE of the posterior, and the rest is the sampling error. Note that the Kalman Gain is optimal since $\omega_{g}=\omega_{r}$.

Now, let us take the different memory scales in the model error into account. These lead to different variances of the prior ensemble mean and the truth: ${\bar{x}}_{N_{e}}^{t,b}∼𝒩(\mu^{t,b},{\beta^{t}}^{2}\;/N_{e})$, and $x^{t,b}∼𝒩(\mu^{t,b},{B^{t}}^{2})$. Thus, we have a slightly different expression for the prior MSE:

(36) $$E_{x^{t,r}}\left[{\left({\bar{x}}_{N_{e}}^{t,b}-x^{t,b}\right)}^{2}\right]=\frac{{\beta^{t}}^{2}}{N_{e}}+{B^{t}}^{2}.$$

Comparing with the expression shown in (.34), if we increase the ensemble size to infinity, the prior MSE is the same; it is just the variance of the truth. But the sampling error part is different. As for the posterior MSE in this scenario:

(37) $$\begin{array}{ll} E_{x^{t,r}}\left[{\left({\bar{x}}_{N_{e}}^{t,a}-x^{t,r}\right)}^{2}\right] & =E_{x^{t,r}}\left[{\left\{{\bar{x}}_{N_{e}}^{t,b}+K_{\omega_{g}}^{x,t}(\bar{y}-H{\bar{x}}_{N_{e}}^{\tau ,r})-x^{t,r}\right\}}^{2}\right]\;\\ & ={B^{t}}^{2}+{\left(K_{\omega_{g}}^{x,t}\right)}^{2}({B^{\tau }}^{2}+r^{2})\;\\ & \;-2K_{\omega_{g}}^{x,t}\text{Cov}(x^{t,r},x^{\tau ,r})\;\\ & \;+\frac{{\beta^{t}}^{2}}{N_{e}}-K_{\omega_{g}}^{x,t}\text{Cov}({\bar{x}}_{N_{e}}^{t,b},{\bar{x}}_{N_{e}}^{\tau ,b}) \end{array}$$

In this case the Kalman Gain $K_{\omega_{g}}^{x,t}$ is not optimal.

Lastly, let us consider the most different case, when we have no knowledge of both the model error memory and its mean. In this case, we also have a bias in the mean of the prior ensemble members besides incorrect variance: ${\bar{x}}_{N_{e}}^{t,b}∼𝒩({\tilde{\mu }}^{t,b},{\beta^{t}}^{2}/N_{e})$. Then, the prior MSE of the ensemble mean has a similar expression as in (28):

(38) $$E_{x^{t,r}}\left[{\left({\bar{x}}_{N_{e}}^{t,b}-x^{t,r}\right)}^{2}\right]=\frac{{\beta^{t}}^{2}}{N_{e}}+{B^{t}}^{2}+{\left(\mu^{t,b}-{\tilde{\mu }}^{t,b}\right)}^{2}.$$

In this scenario, extra errors come from the bias in the mean. The posterior MSE becomes:

(39) $$\begin{array}{ll} & E_{x^{t,r}}\left[{\left({\bar{x}}_{N_{e}}^{t,a}-x^{t,r}\right)}^{2}\right]\;\\ & =E_{x^{t,r}}\left[{\left\{{\bar{x}}_{N_{e}}^{t,b}+K_{\omega_{g}}^{x,t}(\bar{y}-H{\bar{x}}_{N_{e}}^{\tau ,t})-x^{t,r}\right\}}^{2}\right]\;\\ & ={B^{t}}^{2}+{\left(K_{\omega_{g}}^{x,t}\right)}^{2}({B^{\tau }}^{2}+r^{2})-2K_{\omega_{g}}^{x,t}\text{Cov}(x^{t,r},x^{\tau ,r})\;\\ & \;+\frac{{\beta^{t}}^{2}}{N_{e}}-K_{\omega_{g}}^{x,t}\text{Cov}({\bar{x}}_{N_{e}}^{t,b},{\bar{x}}_{N_{e}}^{\tau ,b})+{\left(K_{\omega_{g}}^{x,t}\right)}^{2}{\left(\nu^{t}-{\tilde{\mu }}^{t,b}\right)}^{2}. \end{array}$$

The posterior MSE in this scenario contains the errors that are introduced by the sampling error, incorrect auto‐correlation time‐scale, and the bias term.
